# NT‐proBNP as a Predictor of Atrial Fibrillation Recurrence After Cryoballoon, Radiofrequency and Pulsed‐Field Ablation

**DOI:** 10.1111/pace.70237

**Published:** 2026-04-03

**Authors:** Johannes Bruss, Thomas Kueffer, Hildegard Tanner, Fabian Noti, Andreas Haeberlin, Gregor Thalmann, Nikola Asenov Kozhuharov, Boldizsar Kovacs, Valon Spahiu, Claudia Herrera Siklody, Tobias Reichlin, Laurent Roten

**Affiliations:** ^1^ Department of Cardiology Inselspital, Bern University Hospital, University of Bern Bern Switzerland

**Keywords:** atrial fibrillation, cryoballoon ablation, outcome, pulsed‐field ablation, pulmonary vein isolation, radiofrequency ablation

## Abstract

**Introduction:**

Natriuretic peptides have shown to predict atrial fibrillation (AF) ablation success using thermal energies. Pulsed‐field ablation (PFA) is a novel AF ablation technology. This study investigates the predictive value of NT‐proBNP and clinical parameters for pulmonary vein isolation (PVI) success using PFA, cryoballoon ablation (CBA), and radiofrequency ablation (RFA).

**Methods:**

We included all patients undergoing primary PVI between May 2019 and December 2023 from our prospective registry, excluding those in randomized trials or without NT‐proBNP measured ≤1 day before the procedure. Follow‐up included 7‐day Holter‐ECGs at 3, 6, and 12 months. The primary endpoint was atrial arrhythmia recurrence between days 90–365. NT‐proBNP was dichotomized using the age‐ and sex‐adjusted 95th percentile in healthy adults.

**Results:**

Among 1,391 patients (median age 67 years; 29% female) undergoing primary PVI with RFA (*n* = 302), CBA (*n* = 399), or PFA (*n* = 690), elevated NT‐proBNP levels were observed in 55%, with no significant differences between ablation modalities. NT‐proBNP correlated with age and left atrial volume and was higher in patients with persistent AF. Overall, 1‐year arrhythmia recurrence rate was 32.1%, without differences between ablation technologies. Elevated NT‐proBNP was associated with recurrence across all subgroups. In multivariate Cox regression, NT‐proBNP and persistent AF were the strongest independent predictors of recurrence. A simplified model including only NT‐proBNP and AF type yielded a concordance index of 0.62.

**Conclusions:**

NT‐proBNP is a robust predictor of atrial arrhythmia recurrence after PVI, independent of the ablation technology, and may support shared, evidence‐based decision‐making with patients regarding ablation success probability.

**Trial registration:**

The Swiss‐AF‐PVI registry is registered with ClinicalTrials.gov (NCT03718364)

## Graphical abstract

Central Illustration: In a study investigating AF recurrence after PVI with RFA, CBA, and PFA, elevated NT‐proBNP emerged as a strong predictor of recurrence within the first year, outperforming other clinical measures. Color‐coded recurrence‐free survival probability charts derived from these data are proposed to support individualized clinical decision‐making.

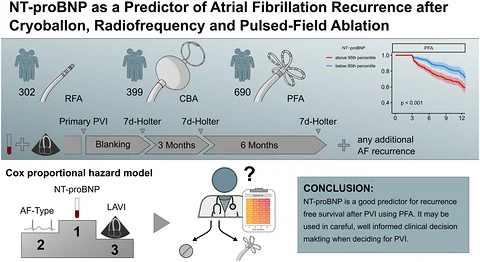

**Abbreviations**
AF: atrial fibrillation
CBA: cryoballoon ablation
eGFR: estimated glomerular filtration rate
LAVI: left atrial volume index
LVEF: left ventricular ejection fraction
NT‐proBNP: *n*‐terminal pro‐B type natriuretic peptide
PFA: pulsed‐field ablation
PV: pulmonary vein
PVI: pulmonary vein isolation
RFA: radiofrequency ablation

## 1 Introduction

Current guidelines have expanded the indication for PVI in patients with both paroxysmal and persistent atrial fibrillation (AF), and the number of procedures continues to increase [1]. In recent years, pulsed‐field ablation (PFA) has gained broad acceptance for pulmonary vein isolation (PVI), due to its procedural efficiency and favorable safety profile compared to thermal ablation technologies [2]. Contemporary ablation technologies, such as cryoballoon ablation (CBA), radiofrequency ablation (RFA), and PFA all consistently achieve acute PVI [3, 4]. Nevertheless, a substantial proportion of patients continues to experience recurrence of AF after PVI [5]. Predicting intermediate‐ and long‐term procedural success remains challenging, as multiple mechanisms beyond pulmonary vein reconnection have been implicated in AF recurrence [6].

Various strategies have been developed to improve candidate selection for successful AF ablation, including advanced electrocardiographic or imaging diagnostics, and clinical scoring systems [7, 8, 9, 10, 11, 12]. Despite these efforts, none of these approaches have achieved widespread adoption in routine clinical practice. Instead, AF type and left atrial volume remain the primary parameters used to estimate AF ablation success [12]. Among biomarkers, the natriuretic peptides have emerged as the most promising candidates for AF screening, disease progression prediction, and estimation of arrhythmia recurrence risk following PVI [13, 14, 15, 16]. Prior studies have primarily evaluated the predictive value of natriuretic peptides following PVI using RFA or CBA. Its prognostic value after PVI with PFA remains unexplored. This study evaluates the value of *N*‐terminal pro‐B‐type natriuretic peptide (NT‐proBNP) for predicting AF recurrence across ablation technologies and compares its performance to commonly used clinical parameters.

## 2 Materials and Methods

### 2.1 Patient Selection

All AF ablation patients are enrolled in our prospective AF ablation registry. For this analysis, we included all patients undergoing primary PVI between April 2019 and December 2023. Patients with missing NT‐proBNP values or incomplete follow‐up were excluded from the study. Patients enrolled in prospective, randomized controlled trials using implantable cardiac monitors (PIFPAF‐PFA, NCT05986526; COMPARE‐CRYO, NCT04704986; SINGLE SHOT CHAMPION, NCT05534581) were also excluded. Inclusion and exclusion criteria for these studies are available in the supplementary material. The registry was approved by the local ethics committee and conducted in accordance with the Declaration of Helsinki. All patients provided written informed consent after being informed about data use. The authors had full access to the dataset and assume responsibility for its accuracy.

### 2.2 Ablation Procedures

Prior to ablation, intracardiac thrombi were excluded and atrial anatomy assessed by cardiac computed tomography angiography and/or transoesophageal echocardiography. Left atrial volume and left ventricular ejection fraction (LVEF) were assessed by 2D transthoracic echocardiography using the Simpsons biplane method. Deep conscious sedation was achieved with propofol, midazolam, and fentanyl according to an operator‐directed, nurse‐administered protocol [17]. Patients at high risk for sedation‐related complications received general anaesthesia. Left atrial access was obtained via fluoroscopy‐guided transseptal puncture. PVI was performed according to standard ablation protocols. Cavotricuspid isthmus ablation was performed at the operator's discretion. The following ablation technologies were used:

#### 2.2.1 RFA

RF ablation was performed using the validated CLOSE protocol, aiming for contiguous, optimized point‐by‐point lesions for PVI. [18] An open‐irrigated RF catheter (ThermoCool SmartTouch SurroundFlow, Biosense Webster, Irvine, CA, USA) and a steerable sheath (Agilis NXT or Destino Reach) were used. Wide antral PVI was performed without additional LA ablation, targeting an ablation index of ≥400 posteriorly and ≥550 anteriorly, with <6 mm interlesion distance and ≥10 g contact force. Power settings were 25 W (posterior) and 35 W (anterior).

#### 2.2.2 CBA

After successful LA access, the standard transseptal sheath was replaced with a steerable sheath (Polarsheath or FlexCath). The cryoballoon (PolarX, Boston Scientific, Menlo Park, CA, USA or Arctic Front, Medtronic, Minneapolis, MN, USA) was positioned at each PV ostium to achieve occlusion. Effective freezes—defined as PV signal elimination or reaching −50°C (PolarX) / −4°C (Arctic Front) within 60 s—were followed by 2 min of additional ablation (“time‐to‐effect plus 2 min strategy”). Ineffective freezes prompted repositioning and reapplication.

#### 2.2.3 PFA

To prevent vagal responses, 0.5 mg of atropine was administered post‐transseptal access. The pentaspline PFA catheter (Farawave, Boston Scientific, Menlo Park, CA, USA) was introduced into the LA via a 13‐F deflectable sheath (Faradrive, Boston Scientific). A 31 mm or 35 mm ablation catheter, selected at operator's discretion, was advanced over a J‐tip guidewire to each PV ostium. PVI was performed using the standard protocol of ≥4 applications per PV in both basket and flower configurations (≥32 total). Additional applications were delivered as needed based on LA anatomy or in case of acute PV reconnection.

### 2.3 Laboratory Analysis

Routine laboratory testing was performed prior to the ablation procedure (the day before or on the same day) and included NT‐proBNP and serum creatinine measurements. All tests were conducted in the hospital's central laboratory using standardized assays (Roche Elecsys proBNP II, Basel, Switzerland). NT‐proBNP was considered normal if below the age‐ and sex‐adjusted 95th percentile for healthy adults, as described by Welsh et al. [19] Reference thresholds are listed in the Table S1. Glomerular filtration rate was calculated from serum creatinine using the 2021 CKD‐EPI equation [20].

### 2.4 Heart Failure History and Heart Rhythm at Admission

For patients recruited after April of 2020, history of heart failure as well as heart rhythm at time of NT‐proBNP measurement were recorded. Heart failure was classified according to the 2021 ESC guidelines for the diagnosis and treatment of acute and chronic heart failure. [21] Heart failure with reduced ejection fraction (HFrEF) and mildly reduced ejection fraction (HFmrEF) were retrospectively assigned to patients with a LVEF ≤40% and 41%–49%, respectively. Heart failure with preserved ejection fraction (HFpEF) was defined by the presence of documented heart failure symptoms, LVEF ≥50%, and either elevated natriuretic peptide levels or structural cardiac abnormalities. Elevated natriuretic peptides were defined as NT‐proBNP >125 pg/mL in sinus rhythm or >365 pg/mL in the presence of AF. Structural abnormalities were defined as an indexed left atrial volume >35 mL/m^2^ in sinus rhythm or >40 mL/m^2^ in the presence of AF.

### 2.5 Echo Measurement and Scores

Left atrial volume index (LAVI) was assessed by transthoracic echocardiography prior to PVI, using the biplane Simpson's method indexed to body surface area. Left atrial diameter was measured in the parasternal long axis view, and LVEF was measured using the biplane Simpson's method or estimated at the sonographer's discretion.

### 2.6 Follow‐Up

Follow‐up included clinical evaluation and 7‐day Holter ECG monitoring at 3, 6, and 12 months post‐procedure (Figure S1a). Follow‐up was conducted either at the study center or by referring cardiologists. Patients were instructed to promptly seek medical attention and obtain an ECG if experiencing symptoms suggestive of arrhythmia recurrence. Antiarrhythmic drugs were not routinely prescribed post‐procedure. Beta‐blockers were continued when indicated for comorbidities such as heart failure, coronary artery disease, or hypertension.

### 2.7 Primary Endpoint

The primary endpoint was atrial arrhythmia recurrence within 12 months post‐procedure, after a blanking period of 90 days. Atrial arrhythmia was defined as any episode of AF, atrial flutter or atrial tachycardia lasting ≥30 s on Holter or other ECG recordings during follow‐up [22].

### 2.8 Statistical Analysis

All statistical Analysis was performed in R, version 4.1.2 [23]. The age‐ and sex‐adjusted 95th percentile in healthy individuals was used as cut‐off for normal NT‐proBNP [19]. Categorical variables were reported as counts and percentages; continuous variables as medians with interquartile ranges (Q1, Q3) or means ± standard deviation, depending on distribution. Depending on variable distribution and sample size, Fisher's exact test, the Kruskal–Wallis test, or the pairwise Wilcoxon test was applied. *P*‐values from pairwise comparisons were adjusted using the Bonferroni‐Holm correction, as applicable. Survival differences were assessed using the Gehan–Wilcoxon test. Multivariate Cox proportional hazards models were used to evaluate the impact of continuous and categorical risk factors on recurrence‐free survival. NT‐proBNP values were log‐transformed due to right‐skewed distribution and to meet proportional hazards assumptions in Cox regression (Figure S2). Predictive performance was further assessed using time‐dependent receiver operating characteristic (ROC) analysis based on the Kaplan–Meier method. Correlations were assessed using Spearman's rank correlation coefficient. Results with a *p*‐value ≤ 0.05 were considered statistically significant.

## 3 Results

### 3.1 Patient and Procedural Characteristics

A total of 1,738 records of patients who underwent primary PVI were extracted from the AF ablation registry during the study period. Patients enrolled in prospective randomized studies using implantable loop recorders (*n* = 268), with missing NT‐proBNP data (*n* = 18), or without follow‐up data (*n* = 61) were excluded. The final cohort comprised 1,391 patients (median age: 67 years; 29% women). PVI was performed using RFA in 302 patients, CBA in 399, and PFA in 690 (Figure S1b).

Baseline characteristics are summarized in Table 1. Persistent AF was more prevalent and left atrial volumes were larger in those treated with PFA compared to those treated with RFA and CBA. Procedural differences among the three ablation groups are detailed in Table 2.

**TABLE 1:** Baseline patient characteristics.

	Total (*n* = 1,391)	RFA (*n* = 302)	CBA (*n* = 399)	PFA (*n* = 690)	*p*‐value
Age (years)	67 (59, 74)	66 (57, 73)	67 (58, 73)	68 (60, 75)	0.013
Women (%)	403 (29)	94 (31)	115 (29)	194 (28)	0.628
BMI (kg/m^2^)	27 (24, 31)	27 (24, 31)	27 (24, 31)	27 (24, 31)	0.957
Persistent AF, *n* (%)	656 (47)	113 (37)	171 (43)	372 (54)	< 0.001
LVEF (%)	60 (50, 60)	60 (50, 60)	60 (53, 60)	60 (50, 60)	0.052
LAVI (ml/m^2^)	39 (32, 48)	37 (30, 46)	38 (30, 47)	41 (34, 51)	< 0.001
Arterial hypertension (%)	789 (58)	161 (53)	234 (59)	394 (59)	0.218
Diabetes mellitus (%)	165 (12)	38 (13)	42 (11)	85 (13)	0.534
Hypercholesterinemia (%)	476 (43)	78 (40)	98 (39)	300 (45)	0.217
Prior stroke (%)	49 (4)	9 (5)	11 (4)	29 (4)	0.982
Prior TIA (%)	36 (3)	8 (4)	8 (3)	20 (3)	0.730
Established OAK (%)	1052 (95)	177 (92)	235 (94)	640 (96)	0.054
CHA_2_DS_2_‐VA (Pts.)	2 (1, 3)	2 (1, 3)	2 (1, 3)	2 (1, 3)	0.238
NYHA (Class)	2 (1, 2)	2 (1, 2)	2 (1, 2)	2 (1, 2)	0.071
NT‐proBNP (pg/ml)	410 (141, 1040)	408 (125, 989)	372 (131, 940)	472 (157, 1111)	0.065
eGFR (ml/min)	88 (73, 97)	91 (77, 99)	90 (76, 97)	85 (71, 96)	< 0.001

*Note*: Shown are counts with percentages or medians with interquartile ranges (first quartile, third quartile).

Abbreviations: AF, atrial fibrillation; BMI, body mass index; CBA, cryoballoon ablation; eGFR, estimated glomerular filtration rate; LAVI, left atrial volume index; LVEF, left ventricular ejection fraction; PFA, pulsed‐field ablation; RFA, radio frequency ablation; TIA, transient ischemic attack.

**TABLE 2:** Procedural characteristics.

	Total (*n* = 1,391)	RFA (*n* = 302)	CBA (*n* = 399)	PFA (*n* = 690)	*p*‐value
Procedure duration (min)	87 (63, 128)	168 (128, 204)	77 (61, 98)	76 (55, 103)	< 0.001
Fluoroscopy time (min)	16.0 (11.0, 23.2)	6.4 (3.0, 12.4)	18.0 (13.1, 23.7)	18.4 (13.6, 5.7)	< 0.001
RFA time (min)	—	37 (30, 47)	—	—	—
Freeze time (min)	—	—	15.7 (13, 20.2)	—	—
PFA applications (*n*)	—	—	—	40 (34, 50)	—

*Note*: Shown are medians with interquartile ranges (first quartile, third quartile).

Abbreviations: CBA, cryoballoon ablation; PFA, pulsed‐field ablation; RFA, radiofrequency ablation.

For a total of 1109 patients heart failure history and hear rhythm at admission was available. Of these patients 652 presented in AF at admission (59%), 438 (39%) had a history of heart failure (Table S2). Standardized mean differences of age, sex, BMI, type of AF, arterial hypertension, diabetes mellitus, prior stroke or TIA, CHAD2DS2VA score, NYHA class as eGFR and NT‐proBNP of this subgroup were below 0.043.

### 3.2 Association of NT‐proBNP Levels and Patient Characteristics

**FIGURE 1:**
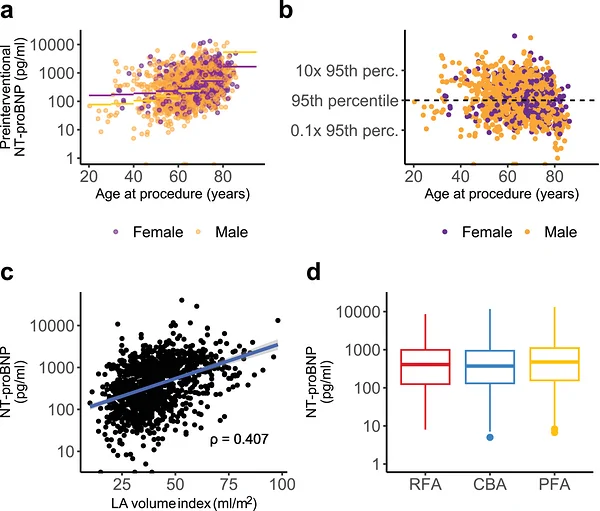
Relation of NT‐proBNP to age, sex and left atrial volume. (a) NT‐proBNP by age and sex. The horizontal lines show the age‐ and sex‐adjusted 95^th^ percentile of NT‐proBNP in a healthy population. (b) NT‐proBNP measurements relative to their respective age and sex adjusted 95^th^ percentile. (c) Relationship of indexed left atrial volume to NT‐proBNP with a linear fit. (d) Boxplot showing the NT‐proBNP‐levels according to ablation technology used. CBA, cryoballoon ablation; PFA, pulsed‐field ablation; RFA, radiofrequency ablation. [Colour figure can be viewed at wileyonlinelibrary.com]

Elevated NT‐proBNP levels—defined as values above the age‐ and sex‐adjusted 95th percentile—were observed in 770 patients (55%; Figure 1b), with no significant differences between ablation groups. NT‐proBNP levels correlated with age (*ρ* = 0.35, *p* < 0.001; Figure 1a) and indexed left atrial volume (*ρ* = 0.41, *p* < 0.001, Figure 1c). Patients with persistent AF had significantly higher NT‐proBNP levels than those with paroxysmal AF (median 820 [83 – 1458] pg/mL vs. 215 [93 – 492] pg/mL; *p* < 0.001). In the subgroup with known heart failure history and heart rhythm at admission, NT‐proBNP was significantly elevated in Patients with a history of heart failure (785 [314, 1484] pg/mL vs. 228 [97, 613] pg/mL, *p* < 0.001). As well as in patients in atrial arrhythmia at the time of NT‐proBNP measurement (963 [473, 1580] pg/mL vs. 190 [97, 490] pg/mL, *p* < 0.001, Figure S3).

### 3.3 Association of NT‐proBNP Levels and Arrhythmia Recurrence

**FIGURE 2:**
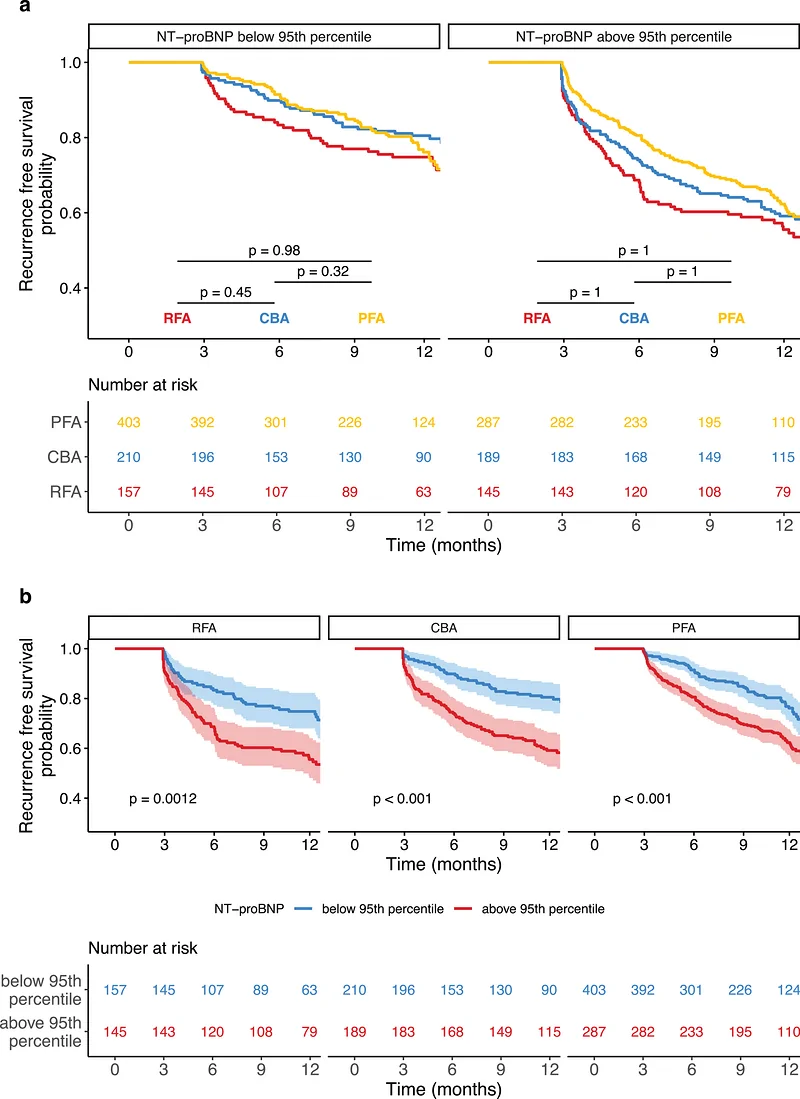
The AF recurrence by NT‐proBNP and modality. Estimated atrial arrhythmia‐free survival for patients with NT‐proBNP <95^th^ and >95^th^ percentile by ablation technology. (a) Kaplan‐Meier curves showing no significant difference between ablation methods in neither the normal nor the elevated NT‐pro‐BNP population. (b) Kaplan‐Meier curves showing a significant difference between elevated and normal NT‐proBNP groups in all ablation technology groups. CBA, cryoballoon ablation; PFA, pulsed‐field ablation; RFA, radiofrequency ablation. [Colour figure can be viewed at wileyonlinelibrary.com]

Kaplan‐Meier analysis showed a 1‐year AF recurrence rate of 32.1% in the overall cohort. No significant difference in recurrence was observed between ablation modalities (RFA: 35.2%; CBA: 30.7%; PFA: 32.1%; RFA vs. CBA: *p* = 0.471; RFA vs. PFA: *p* = 0.722; CBA vs. PFA: *p* = 0.471). Stratified analyses by NT‐proBNP subgroup (above vs. below cutoff) revealed no significant difference in recurrence rates between ablation modalities (Figure 2a). NT‐proBNP above the adjusted 95th percentile was associated with atrial arrhythmia recurrence in the overall cohort (*p* < 0.001) and within each ablation subgroup (Figure 2b).

### 3.4 Predictive Modelling

**FIGURE 3:**
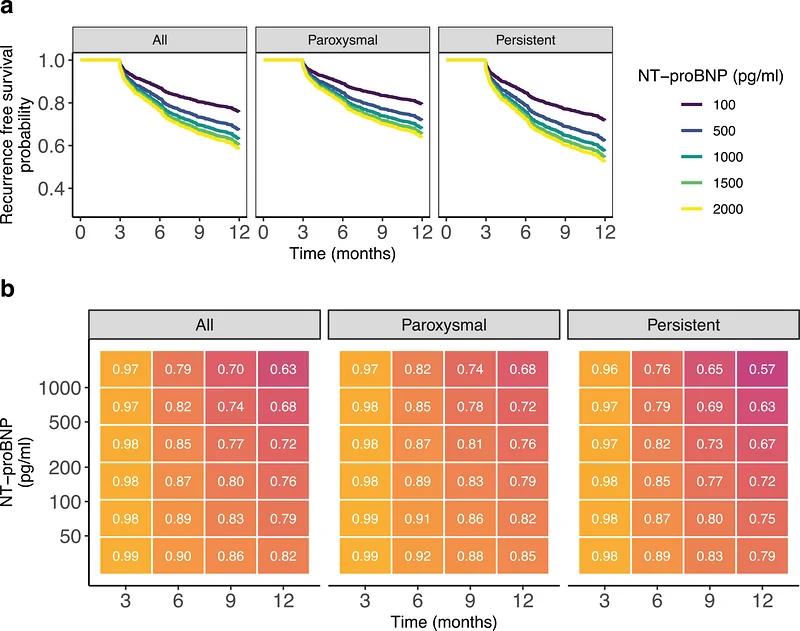
Predicting arrhythmia recurrence using NT‐proBNP. (a) Stratified Kaplan‐Meier curves derived from a multivariate Cox proportional hazard model fitted on NT‐proBNP and type of AF (paroxysmal or persistent). (b) Color‐coded survival probability chart showing the expected atrial arrhythmia‐free survival over time by NT‐proBNP and type of AF (paroxysmal or persistent). Example: A patient with a NT‐proBNP of ∼ 300 pg/ml and persistent AF is represented on the third line on the right panel, resulting in an estimated probability of atrial arrhythmia‐free survival after 12 Months of 72%. CBA, cryoballoon ablation; PFA, pulsed‐field ablation; RFA, radiofrequency ablation. [Colour figure can be viewed at wileyonlinelibrary.com]

**FIGURE 4:**
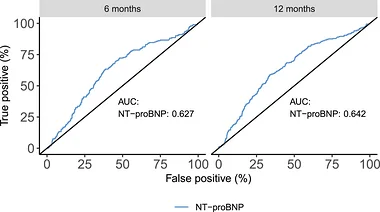
*ROC curves*. ROC of NT‐proBNP at 6 and 12 Months after the procedure. [Colour figure can be viewed at wileyonlinelibrary.com]

Univariate and multivariate Cox proportional hazards models were used to identify predictors of AF recurrence, including log‐transformed NT‐proBNP, ablation technology, age, sex, BMI, eGFR, LAVI, and AF type (paroxysmal vs. persistent). In the multivariate model, NT‐proBNP was the strongest predictor of arrhythmia recurrence (HR 1.50, *p* < 0.001), followed by persistent AF (HR 1.39, *p* = 0.006). Additional predictors included BMI, indexed LAVI, and sex. Ablation modality, age, and renal function were not independently associated with recurrence (Table 3). A simplified model including only NT‐proBNP and AF type was fitted; stratified recurrence‐free survival probabilities are shown in Figure 3a. The model showed a concordance index of 0.62 ± 0.014. In the subgroup with known rhythm at admission or heart failure history, adding heart failure history to this model did not improve outcome prediction (HR 0.73 – 1.14, *p* = 0.4). Adding heart rhythm at admission to the model did not improve concordance nor alter the influence of NT‐proBNP on the outcome (Table S3). Color‐coded survival probability matrices derived from the simplified model are presented in Figure 3b. ROC‐Curves of prediction of AF‐recurrence by NT‐proBNP at 6 and 12 months are shown in Figure 4.

**TABLE 3:** Cox proportional hazard model.

	HR	95% confidence interval	*p*‐value
**Univariate Models**			
NT‐proBNP (pg/ml, log transformed)	1.91	1.62–2.24	< 0.001
Age (years)	1.01	1–1.02	0.025
Male sex	0.78	0.65–0.95	0.012
LAVI (ml/m^2^)	1.02	1.01–1.02	< 0.001
Persistent AF	1.83	1.53–2.2	< 0.001
BMI (kg/m^2^)	1.03	1.01–1.05	< 0.001
eGFR (ml/min)	1	0.99–1	0.059
PFA performed	1.04	0.86–1.25	0.709
RFA performed	1.127	0.91–1.38	0.295
**Multivariate model**			
NT‐proBNP (pg/ml, log transformed)	1.50	1.19–1.89	< 0.001
Age (years)	0.99	0.99–1.01	0.981
Male sex	0.79	0.64–0.99	0.041
LAVI (ml/m^2^)	1.01	1.00–1.02	0.038
Persistent AF	1.39	1.10–1.75	0.006
BMI (kg/m^2^)	1.02	1.00–1.04	0.037
eGFR (ml/min)	1.00	0.99–1.01	0.900
PFA performed	1.01	0.79–1.28	0.955
RFA performed	1.27	0.98–1.64	0.071
**Model used for colour coded survival probability chart**			
NT‐proBNP (pg/ml log transformed)	1.67	1.40–2.00	< 0.001
Persistent AF	1.44	1.18–1.76	< 0.001

*Note*: Results of Cox proportional hazard models.

Abbreviations: AF, atrial fibrillation; BMI, body mass index; eGFR, estimated glomerular filtration rate, LAVI, left atrial volume index; PFA, pulsed‐field ablation; RFA, radiofrequency ablation.

## 4 Discussion

In this large, real‐world cohort of patients undergoing first‐time PVI for AF

1. More than half (55%) exhibited elevated NT‐proBNP levels above the age‐ and sex‐adjusted 95th percentile
2. NT‐proBNP was a significant predictor of arrhythmia recurrence irrespective of the ablation technology used, including RFA, CBA, and PFA
3. The combination of NT‐proBNP and AF type yielded a simplified, clinically applicable model for predicting ablation success

These findings underscore the potential value of NT‐proBNP as an accessible biomarker for patient selection and shared decision‐making in contemporary AF ablation practice. Elevated NT‐proBNP levels are frequent among patients undergoing AF ablation, especially in those with persistent AF. This observation is consistent with previous studies, suggesting that increased left atrial wall stress may drive both NT‐proBNP elevation and AF susceptibility, independent of left ventricular function [14, 15, 24, 25].

Recurrence rates observed in our study were consistent with those reported in previous prospective and registry‐based studies across all three ablation modalities [2, 26, 27, 28]. Previous studies have identified elevated NT‐proBNP and BNP levels as reliable predictors of AF recurrence after PVI with CBA and RFA [15, 29, 30]. Our findings confirm these associations for thermal ablation and extend them to PFA.

Among the clinical variables evaluated, pre‐procedural NT‐proBNP demonstrated the strongest predictive value for one‐year arrhythmia‐free survival, followed by AF type—a relationship previously reported for CBA by several studies [15, 31]. While previous studies have identified the combination of LAVI and AF type as a useful predictor of arrhythmia recurrence, our findings suggest that NT‐proBNP combined with AF type offers superior prognostic value [12]. Furthermore, when using NT‐proBNP as the main predictor of PVI outcome, heart failure history did not influence the prediction and heart rhythm at the time of NT‐proBNP measurement did not perform better than AF type. The superior predictive value of NT‐proBNP over LAVI may be explained by its ability to reflect the overall burden of atrial strain, including mechanisms not associated with left atrial dilation, such as inflammation. A small number of studies have investigated these relationships. [32, 33, 34] However, large scale data remains rare and underscores the need for further investigation into the pathophysiological mechanisms driving NT‐proBNP elevation. Supporting this notion, studies on other atrial disease markers—such as MRI‐detected atrial fibrosis—suggest that left atrial dilation is not the sole indicator of pro‐arrhythmic atrial remodelling [35]. It has to be noted however that overall concordance of the final model remained low, emphasising the remaining variability of PVI outcome, even when using NT‐proBNP and AF type as predictors.

Interestingly, regression analysis suggested that male sex was associated with a more favorable outcome following PVI. However, this finding should be interpreted with caution. The retrospective and observational design of the study may have introduced selection bias, for example if women presented at a later stage of disease and with more advanced atrial remodeling. In addition, known sex‐related differences in NT‐proBNP concentrations may have influenced this association and should be considered when interpreting the results. Our findings help generate hypothesis for future clinical research. Elevated NT‐proBNP levels may identify patients in whom adjunctive ablation strategies beyond primary PVI or alternative strategies like “pace and ablate” could be beneficial. This approach is currently under investigation in randomized prospective clinical trials in patients with persistent AF. An additional advantage of NT‐proBNP in this application is its broad clinical availability, routine use in heart failure diagnostics, relatively low cost compared to advanced imaging modalities such as cardiac MRI, and its independence from operator variability, in contrast to echocardiographic assessments [36].

Current European guidelines recommend first‐line rhythm control with PVI as the primary therapy for all patients with symptomatic paroxysmal AF, selected patients with persistent AF, and those with AF‐related complications such as tachycardia‐induced cardiomyopathy or tachycardia‐bradycardia syndrome [1]. However, due to limited predictive data, the guidelines state that “the optimal ablation strategy has not been clarified in the non‐paroxysmal AF population.” We propose that, once prospectively validated, combining NT‐proBNP with AF type may help address this gap and support individualized clinical decision‐making. To support risk stratification, we advocate the use of intuitive, color‐coded survival probability charts to estimate the likelihood of arrhythmia recurrence. These visual tools may in future aid shared decision‐making, particularly when considering alternative strategies such as pace‐and‐ablate.

### 4.1 Limitations

This study has limitations inherent to its retrospective, single‐center, registry‐based design. Despite structured follow‐up and broad inclusion through the SWISS‐AF‐PVI registry, the observational nature of the study may introduce selection and reporting bias. The potential effect of ongoing heart failure therapy on baseline NT‐proBNP could not be adjusted for, possibly confounding its association with arrhythmia recurrence. Although asymptomatic AF episodes were detected via 7‐day Holter monitoring, the predictive value of NT‐proBNP for symptomatic AF recurrence remains uncertain. Arrhythmia recurrence may also have been underestimated due to intermittent 7‐day Holter monitoring instead of continuous rhythm surveillance via implantable cardiac monitors. Before implementation into clinical practice, the proposed models will require a multicenter prospective external validation.

## 5 Conclusions

NT‐proBNP was a strong, ablation‐technology‐independent predictor of atrial arrhythmia recurrence after PVI. Elevated levels were associated with persistent AF and larger left atrial volume. While recurrence rates did not differ across RFA, CBA, and PFA, NT‐proBNP consistently identified patients at higher risk. A simplified model based on NT‐proBNP and AF type may support shared, evidence‐based decision‐making with patients regarding ablation success probability.

## Funding

This research did not receive any specific grant from funding agencies in the public, commercial, or not‐for‐profit sectors.

## Ethics Statement

The Swiss‐AF‐PVI Registry was approved by the ethics committee of the Canton of Bern, all analysis were performed as outlined in the registries approval (KEK PB_2018_00226).

## Conflicts of Interest

A. Haeberlin: has received travel fees/educational grants from Medtronic, Biotronik, Abbott, and Philips/Spectranetics without impact on his personal remuneration. He serves as a proctor for Medtronic. He has received research grants from the Swiss National Science Foundation, the Swiss Innovation agency Innosuisse, the Swiss Heart Foundation, the University of Bern, the University Hospital Bern, the Velux Foundation, the Hasler Foundation, the Swiss Heart Rhythm Foundation, and the Novartis Research Foundation. He is Co‐founder and clinical head of Act‐Inno AG. L. Roten: has received research grants from Medtronic, the Swiss National Foundation, the Swiss Heart Foundation, the Immanuel and Ilse Straub Foundation and the Sitem Insel Support Fund, all for work outside the submitted study. He has received speaker fees / honoraria from Biosense Webster, Boston Scientific, Abbott and Medtronic. T. Reichlin: Research grants from the Swiss National Science Foundation, the Swiss Heart Foundation, the sitem insel support fund, Biotronik, Boston Scientific and Medtronic, all for work outside the submitted study. He has received speaker/consulting honoraria or travel support from Abbott/SJM, Biosense‐Webster, Biotronik, Boston Scientific and Medtronic. He has received support for his institution's fellowship program from Abbott/SJM, Biosense‐Webster, Biotronik, Boston‐Scientific and Medtronic. N. Kozhuharov: has received research grants from the Swiss National Science Foundation (P400PM‐194477 and P5R5PM_210856), Gottfried und Julia Bangerter‐Rhyner‐Stiftung, Freiwillige Akademische Gesellschaft, L. & Th. La Roche Stiftung and the European Society of Cardiology. F. Noti: Medtronic, Abbott: Travel fees, speaker fees, educational grant; Boston Scientific, Philips Spectranetics: Travel fees, educational grant; Biotronik: Institutional grant all for work outside the submitted study. T. Kueffer: Research grants from the Swiss Heart Foundation for work outside the submitted study. All other authors report no conflicts of interest related to this paper.

## Supporting information




**Supplementary Table 1**: Sex‐ and age‐adjusted 95th percentiles of NT‐proBNP in healthy adults, taken from Welsh et al. 2022.
**Supplementary Table 2**: Patient characteristics in subgroup analysis of Patients with known heart failure history and known heart rhythm on admission Shown are counts with percentages or medians with interquartile ranges (first quartile, third quartile). AF, atrial fibrillation; BMI, body mass index; CBA, cryoballoon ablation; eGFR, estimated glomerular filtration rate; HFmrEF, heart failure with mildly reduced ejection fraction; HFpEF, heart failure with preserved ejection fraction; HFrEF, heart failure with reduced ejection fraction; LAVI, left atrial volume index; LVEF, left ventricular ejection fraction; PFA, pulsed‐field ablation; RFA, radio frequency ablation; SMD, standardized mean difference; TIA transient ischemic attack. * Standardized mean difference was calculated between the total subset and the full cohort.
**Supplementary Table 3**: Comparison of Cox regression models using either type of atrial fibrillation or heart rhythm prior to PVI. Shown are results of cox regression models using NT‐proBNP in combination with either persistent atrial fibrillation, sinus rhythm at NT‐proBNP measurement or both as predictors. The models were fitted on the subset of patients were heart rhythm at time of NT‐proBNP measurement was recorded. Calibration slopes were calculated using bootstrap validation with 200 samples. AF, atrial fibrillation; BIC, Bayesian Information Criterion; HR, hazard ratio; PVI, pulmonary vein isolation; SR, sinus rhythm.
**Supplementary Figure 1**: Follow‐up strategy and study flow chart: a) Laboratory analysis and echo measurements were performed ≤1 day before PVI. Regular follow with 7‐day Holter monitoring was conducted at 3, 6, and 12 months after a 90 day blanking period. Additional AF episodes outside scheduled follow‐up were also recorded as endpoints. b) Of the 2,313 patients enrolled in the SWISS‐AF‐PVI registry at the study center during the study period, 1,738 patients underwent primary PVI. Of these, 1,391 patients were included in the analyses. Patients participating in prospective, randomized trials using an implantable loop recorder were excluded from analysis, as well as patients without NT‐proBNP measurements or follow‐up data. AF: atrial fibrillation; CBA: cryoballoon ablation; ILR: implantable loop recorder; PFA: pulsed‐field ablation; PVI: pulmonary vein isolation; RFA: radio frequency ablation.
**Supplementary Figure 2**: Distribution of NT‐proBNP measurements by ablation method and type of AF, displayed on a linear and logarithmic scale. The black line represents the distribution of all patients receiving PVI with a given ablation method, regardless AF type. AF: atrial fibrillation; CBA: cryoballoon ablation; PFA: pulsed‐field ablation; PVI: pulmonary vein isolation; RFA: radio frequency ablation.
**Supplementary Figure 3**: NT‐proBNP measurements by type of atrial fibrillation and (a) heart rhythm at admission or (b) heart failure history. AF, atrial fibrillation.

## Data Availability

The data that support the findings of this study are available from the corresponding author upon reasonable request.
