# Supplementary material for: NT‐proBNP as a Predictor of Atrial Fibrillation Recurrence After Cryoballoon, Radiofrequency and Pulsed‐Field Ablation
Source: Pacing Clin Electrophysiol. 2026 Apr 3;49(9):1364–73. doi: 10.1111/pace.70237 (PMC13542319; doi:10.1111/pace.70237)
Supplement: Supplementary file 1 [file PACE-49-1364-s001.pdf]

Inclusion and exclusion criteria for the randomized controlled trials using an implantable loop recorder where participants were excluded in this study:

PIFPAF-PFA, NCT05986526:

Inclusion criteria:

- Age 18 years and older, signed informed consent
- Persistent atrial fibrillation documented on a 12 lead ECG, Holter monitor or implantable cardiac device within last 2 years of enrollment
- Persistent atrial fibrillation is defined as a sustained episode lasting > 7 days
- Candidate for ablation based on current atrial fibrillation guidelines
- Continuous anticoagulation with Vitamin-K-Antagonists or a NOAC for ≥4 weeks prior to the ablation; or a transesophageal echocardiography and/or CT scan that excludes left atrial thrombus ≤48 hours before the ablation procedure

Exclusion criteria:

- Previous left atrial ablation or left atrial surgery
- Left atrial diameter >60 mm in the parasternal long axis
- Patients with paroxysmal atrial fibrillation
- Patients with persistent atrial fibrillation lasting >3 years
- AF due to reversible causes (e.g. hyperthyroidism, cardiothoracic surgery)
- Intracardiac thrombus
- Pre-existing pulmonary vein stenosis or pulmonary vein stent
- Pre-existing hemidiaphragmatic paralysis
- Contraindication to anticoagulation or radiocontrast materials
- Prior mitral valve surgery
- Severe mitral regurgitation or moderate/severe mitral stenosis
- Myocardial infarction during the 3-month period preceding the consent date
- Ongoing triple antithrombotic/anticoagulation therapy
- Cardiac surgery during the 3-month interval preceding the informed consent date or scheduled cardiac surgery/ transcatheter aortic valve implantation
- Significant congenital heart defect (including atrial septal defects or
- pulmonary vein abnormalities but not including a patent foramen ovale)
- NYHA class III or IV congestive heart failure
- Left ventricular ejection fraction (LVEF) <35%
- Hypertrophic cardiomyopathy (wall thickness >1.5 cm)
- Significant chronic kidney disease (eGFR <30 ml/min)
- Uncontrolled hyperthyroidism
- Cerebral ischemic event (stroke or TIA) during the 6-month interval preceding the informed consent date
- Ongoing systemic infections
- History of cryoglobulinemia
- Cardiac amyloidosis
- Pregnancy (to exclude pregnancy a blood test (HCG) is performed in women < 50 years before inclusion)
- Life expectancy less than one year per physician opinion
- Currently participating in any other clinical trial, which may confound the results of this trial
- Unwilling or unable to comply fully with the study procedures and follow-up

COMPARE-CRYO, NCT04704986:

Inclusion criteria:

- Age 18 years and older, signed informed consent
- Paroxysmal AF documented on a 12-lead electrocardiogram (ECG) or Holter monitor (lasting ≥30 seconds) within the last 24 months
- Candidates for PVI based on current AF guidelines

Exclusion criteria:

- Previous LA ablation or LA surgery
- AF due to reversible causes (e.g. hyperthyroidism, cardiothoracic surgery)
- Intracardiac thrombus
- Pre-existing PV stenosis or PV stent
- Pre-existing hemidiaphragmatic paralysis
- Contraindication to anticoagulation or radiocontrast materials
- Cardiac valve prosthesis
- Clinically significant (moderately-severe or severe) mitral regurgitation or stenosis
- Myocardial infarction, PCI/ PTCA, or coronary artery stenting during the 3-month period preceding the consent date
- Cardiac surgery during the three-month interval preceding the consent date or scheduled cardiac surgery/TAVI procedure
- Significant congenital heart defect (including atrial septal defects or PV abnormalities but not including PFO)
- NYHA class III or IV congestive heart failure
- Left ventricular ejection fraction (LVEF) <35%
- Hypertrophic cardiomyopathy (wall thickness >1.5 cm)
- Significant chronic kidney disease (eGFR <30 ml/min)
- Uncontrolled hyperthyroidism
- Cerebral ischemic event (stroke or TIA) during the six-month interval preceding the consent date
- Ongoing systemic infections
- History of cryoglobulinemia
- Pregnancy*
- Life expectancy less than one year per physician opinion
- Currently participating in any other clinical trial of a drug, device or biological material during the duration of this study.
- Unwilling or unable to comply fully with study procedures and follow-up.

SINGLE SHOT CHAMPION, NCT05534581:

Inclusion criteria:

- Age 18 years and older, signed informed consent
- Paroxysmal AF documented on a 12-lead electrocardiogram (ECG) or Holter monitor (lasting ≥30 seconds) within the last 24 months
- Candidates for PVI based on current AF guidelines
- Continuous anticoagulation with Vitamin-K-Antagonists or a NOAC for ≥4 weeks prior to the ablation; or a transesophageal echocardiography and/or CT scan that excludes left atrial thrombus ≤48 hours before the ablation procedure

Exclusion criteria:

- Previous left atrial (LA) ablation or LA surgery
- AF due to reversible causes (e.g. hyperthyroidism, cardiothoracic surgery)
- Intracardiac thrombus
- Pre-existing pulmonary vein stenosis or PV stent
- Pre-existing hemidiaphragmatic paralysis
- Contraindication to anticoagulation or radiocontrast materials
- Prior mitral valve surgery
- Severe mitral regurgitation or moderate/severe mitral stenosis
- Myocardial infarction during the 3-month period preceding the consent date
- Ongoing triple therapy
- Cardiac surgery during the three-month interval preceding the consent date or scheduled cardiac surgery/TAVI procedure
- Significant congenital heart defect (including atrial septal defects or PV abnormalities but not including PFO)
- NYHA class III or IV congestive heart failure
- Left ventricular ejection fraction (LVEF) <35%
- Hypertrophic cardiomyopathy (wall thickness >1.5 cm)
- Significant chronic kidney disease (CKD; eGFR <30 ml/min)
- Uncontrolled hyperthyroidism
- Cerebral ischemic event (stroke or TIA) during the six-month interval preceding the consent date
- Ongoing systemic infections
- History of cryoglobulinemia
- Cardiac amyloidosis
- Pregnancy
- Life expectancy less than one (1) year per physician opinion
- Currently participating in any other clinical trial, which may confound the results of this trial.
- Unwilling or unable to comply fully with study procedures and follow-up.

Supplementary Table 1

	95th percentile of NT-proBNP (pg/ml)	
Age range (years)	male	female
< 30	77	162
30-39	82	174
40-49	106	191
50-59	152	229
60-69	233	283
70-79	545	519
≥ 80	5385	1665

Sex- and age-adjusted 95th percentiles of NT-proBNP in healthy adults, taken from Welsh et al. 2022.

Supplementary Figure 1

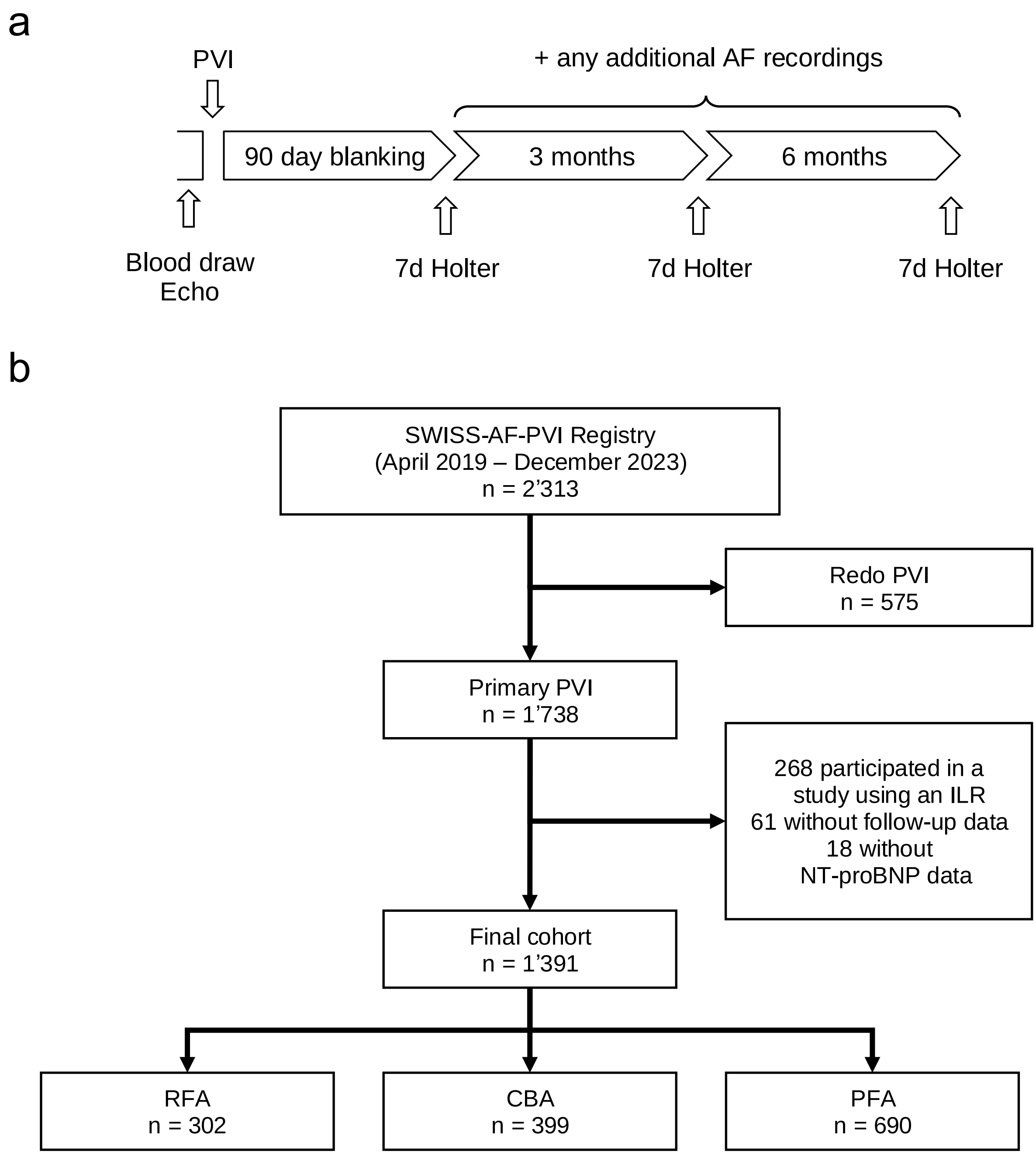

Follow-up strategy and study flow chart: a) Laboratory analysis and echo measurements were performed ≤1 day before PVI. Regular follow with 7-day Holter monitoring was conducted at 3, 6 and 12 months after a 90 day blanking period. Additional AF episodes outside scheduled follow-up were also recorded as endpoints. b) Of the 2’313 patients enrolled in the SWISS-AF-PVI registry at the study centre during the study period, 1’738 patients underwent primary PVI. Of these, 1’391 patients were included in the analyses. Patients participating in prospective, randomized trials using an implantable loop recorder were excluded from analysis, as well as patients without NT-proBNP measurements or follow-up data. AF: atrial fibrillation; CBA: cryoballoon ablation; ILR: implantable loop recorder; PFA: pulsed-field ablation; PVI: pulmonary vein isolation; RFA: radio frequency ablation.

Supplementary Figure 2

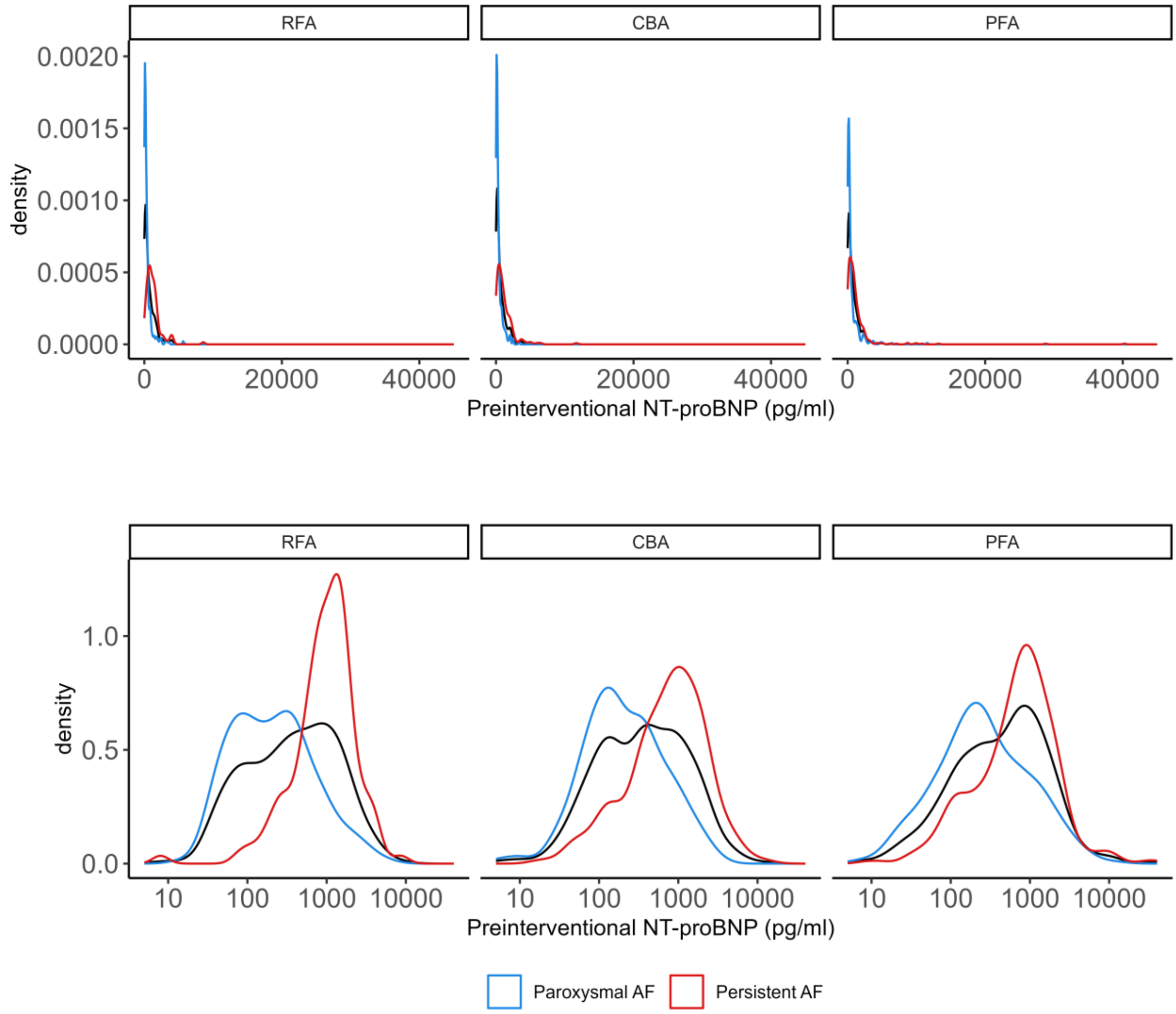

Distribution of NT-proBNP measurements by ablation method and type of AF, displayed on a linear and logarithmic scale. The black line represents the distribution of all patients receiving PVI with a given ablation method, regardless AF type. AF: atrial fibrillation; CBA: cryoballoon ablation; PFA: pulsed-field ablation; PVI: pulmonary vein isolation; RFA: radio frequency ablation.

Supplementary Table 2

	Total (n = 1109)	RFA (n = 193)	CBA (n = 250)	PFA (n = 666)	p-value	SMD*
Age (years)	68 (60, 74)	67 (58, 74)	69 (59, 74)	68 (60, 75)	0.214	0.043
Women (%)	319 (29)	63 (33)	73 (29)	183 (27)	0.372	0.005
BMI (kg/m^2)	27 (24, 31)	27 (24, 31)	27 (24, 31)	27 (24, 31)	0.966	0.004
Persistent AF, n (%)	545 (49)	76 (39)	110 (44)	359 (54)	< 0.001	0.040
LVEF (%)	60 (50, 60)	60 (55, 60)	60 (55, 60)	60 (50, 60)	0.002	0.032
LAVI (ml/m^2)	40 (33, 48)	38 (33, 46)	38 (31, 47)	41 (34, 51)	< 0.001	0.043
Arterial Hypertension (%)	629 (57)	95 (49)	141 (56)	393 (59)	0.054	0.019
Diabetes Mellitus (%)	145 (13)	27 (14)	34 (14)	84 (13)	0.849	0.031
Hypercholesterinemia (%)	475 (43)	78 (40)	98 (39)	299 (45)	0.227	0.001
Prior Stroke (%)	48 (4)	9 (5)	11 (4)	28 (4)	0.961	0.004
Prior TIA (%)	36 (3)	8 (4)	8 (3)	20 (3)	0.732	<0.001
Established OAK (%)	1051 (95)	177 (92)	235 (94)	639 (96)	0.055	<0.001
CHA_2DS_2-VA (Pts.)	2 (1, 3)	2 (1, 3)	2 (1, 3)	2 (1, 3)	0.574	0.022
NYHA (Class)	2 (1, 2)	2 (1, 2)	2 (1, 2)	2 (1, 2)	0.07	<0.001
NT-proBNP (pg/ml)	398 (138, 1036)	355 (109, 989)	330 (120, 847)	456 (152, 1097)	0.023	0.005
eGFR (ml/min)	88 (73, 97)	91 (77, 99)	91 (78, 97)	85 (71, 96)	< 0.001	0.001
Sinus rhythm at admission (%)	652 (59)	122 (63)	168 (67)	362 (54)	< 0.001	-
Heart failure history (%)	509 (37)	98 (32)	122 (31)	289 (42)	< 0.001	-
HFpEF	375 (27)	77 (25)	98 (25)	200 (29)		-
HFmrEF	44 (3)	9 (3)	10 (3)	25 (4)		-
HFrEF	90 (6)	12 (4)	14 (4)	64 (9)		-

Patient characteristics in subgroup analysis of Patients with known heart failure history and known heart rhythm on admission Shown are counts with percentages or medians with interquartile ranges (1st quartile, 3rd quartile). AF, atrial fibrillation; BMI, body mass index; CBA, cryoballoon ablation; eGFR, estimated glomerular filtration rate; HFmrEF, heart failure with mildly reduced ejection fraction; HFpEF, heart failure with preserved ejection fraction; HFrEF, heart failure with reduced ejection fraction; LAVI, left atrial volume index; LVEF, left ventricular ejection fraction; PFA, pulsed-field ablation; RFA, radio frequency ablation; SMD, standardized mean difference; TIA transient ischemic attack. * Standardized mean difference was calculated between the total subset and the full cohort.

Supplementary Figure 3

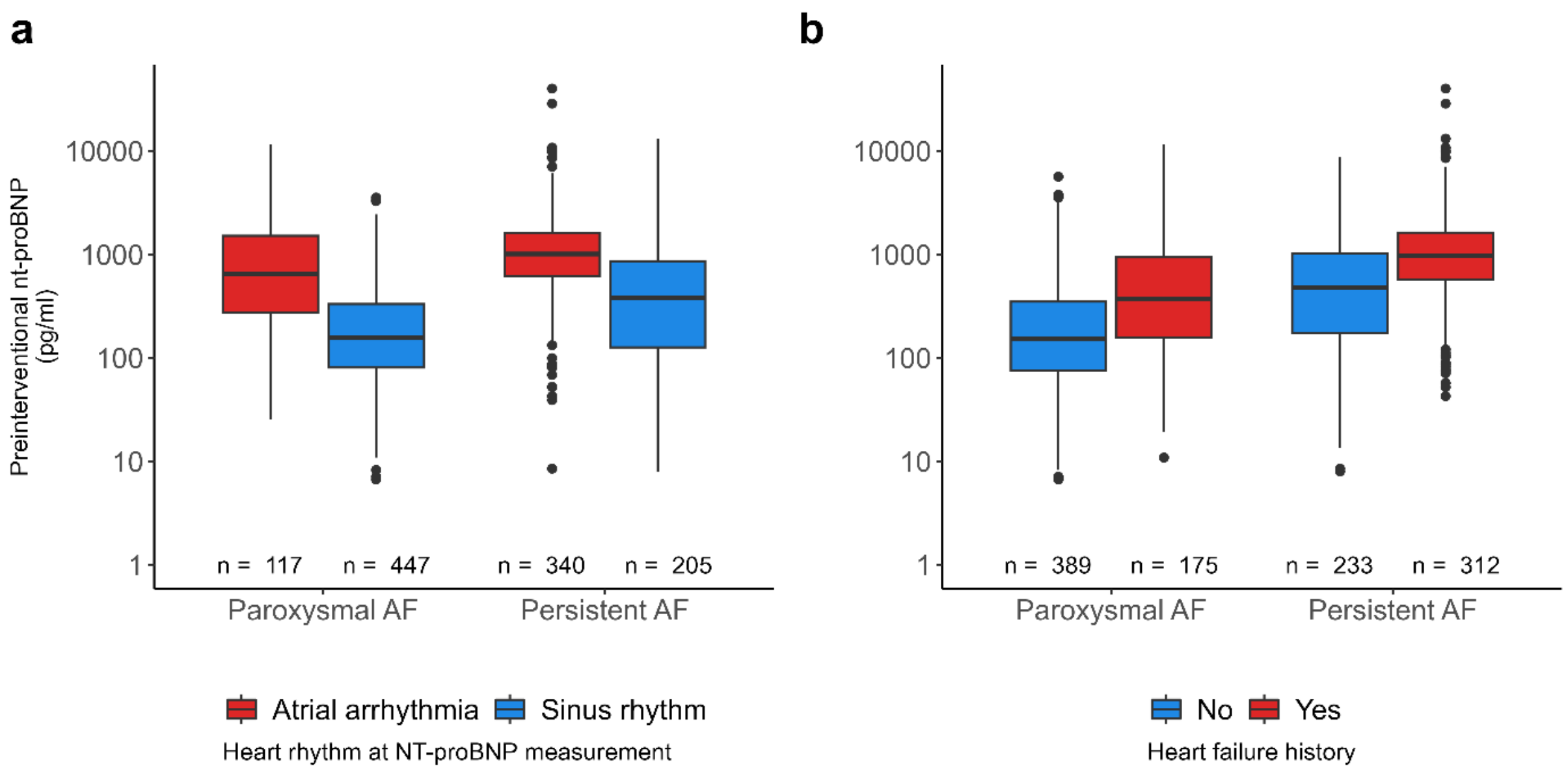

NT-proBNP measurements by type of atrial fibrillation and (a) heart rhythm at admission or (b) heart failure history. AF, atrial fibrillation.

Supplementary Table 3

**Model**	**NT-proBNP + Type of AF**	**NT-proBNP + SR prior to PVI**	**NT-proBNP + Type of AF + SR prior to PVI**
HR NT-proBNP	1.65 (1.36-2.01, p < 0.001)	1.59 (1.30-1.95, p < 0.001)	1.50 (1.21-1.85, p < 0.001)
HR persistent AF	1.41 (1.13 - 1.77, p < 0.005)	-	1.32 (1.04 - 1.66, p = 0.021)
HR SR prior to PVI	-	0.70 (0.55 - 0.88, p < 0.005)	0.75 (0.59 - 0.96, p = 0.021)
Concordance Index	0.616	0.614	0.617
BIC	4755.058	4755.045	4755.632
Slope	1.0079	1.0114	0.9888

Comparison of Cox regression models using either type of atrial fibrillation or heart rhythm prior to PVI. Shown are results of cox regression models using NT-proBNP in combination with either persistent atrial fibrillation, sinus rhythm at NT-proBNP measurement or both as predictors. The models were fitted on the subset of patients were heart rhythm at time of NT-proBNP measurement was recorded. Calibaration slopes were calculated using bootstrap validation with 200 samples. AF, atrial fibrillation; BIC, Bayesian Information Criterion; HR, hazard ratio; PVI, pulmonary vein isolation; SR, sinus rhythm.
